# Replicate exome-sequencing in a multiple-generation family: improved interpretation of next-generation sequencing data

**DOI:** 10.1186/s12864-015-2107-y

**Published:** 2015-11-25

**Authors:** Praveen F. Cherukuri, Valerie Maduro, Karin V. Fuentes-Fajardo, Kevin Lam, NISC Comparative Sequencing Program, David R. Adams, Cynthia J. Tifft, James C. Mullikin, William A. Gahl, Cornelius F. Boerkoel

**Affiliations:** NIH Undiagnosed Diseases Program, Common Fund, Office of the Director, NIH, Bethesda, MD USA; NIH Intramural Sequencing Center, National Human Genome Research Institute, NIH, Bethesda, MD USA; Office of the Clinical Director, National Human Genome Research Institute, NIH, Bethesda, MD USA; Inova Translational Medicine Institute, Inova Health System, Falls Church, VA USA

## Abstract

**Background:**

Whole-exome sequencing (WES) is rapidly evolving into a tool of choice for rapid, and inexpensive identification of molecular genetic lesions within targeted regions of the human genome. While biases in WES coverage of nucleotides in targeted regions are recognized, it is not well understood how repetition of WES improves the interpretation of sequencing results in a clinical diagnostic setting.

**Method:**

To address this, we compared independently generated exome-capture of six individuals from three-generations sequenced in triplicate. This generated between 48x-86x mean target depth of high-quality mapped bases (>Q20) for each technical replicate library. Cumulatively, we achieved 179 - 208x average target coverage for each individual in the pedigree. Using this experimental design, we evaluated stochastics in WES interpretation, genotyping sensitivity, and accuracy to detect *de novo* variants.

**Results:**

In this study, we show that repetition of WES improved the interpretation of the capture target regions after aggregating the data (93.5 - 93.9 %). Compared to 81.2 - 89.6 % (50.2-55.4 Mb of 61.7 M) coverage of targeted bases at ≥20x in the individual technical replicates, the aggregated data covered 93.5 - 93.9 % of targeted bases (57.7 – 58.0 of 61.7 M) at ≥20x threshold, suggesting a 4.3 – 12.7 % improvement in coverage. Each individual’s aggregate dataset recovered 3.4 – 6.4 million bases within variable targeted regions. We uncovered technical variability (2-5 %) inherent to WES technique. We also show improved interpretation in assessing clinically important regions that lack interpretation under current conditions, affecting 12–16 of the 56 genes recommended for secondary analysis by American College of Medical Genetics (ACMG). We demonstrate that comparing technical replicate WES datasets and their derived aggregate data can effectively address overall WES genotyping discrepancies.

**Conclusion:**

We describe a method to evaluate the reproducibility and stochastics in exome library preparation, and delineate the advantages of aggregating the data derived from technical replicates. The implications of this study are directly applicable to improved experimental design and provide an opportunity to rapidly, efficiently, and accurately arrive at reliable candidate nucleotide variants.

**Electronic supplementary material:**

The online version of this article (doi:10.1186/s12864-015-2107-y) contains supplementary material, which is available to authorized users.

## Background

Whole-exome sequencing (WES) is becoming a rapid and cost-effective molecular diagnostic tool in individuals with genetic diseases [1–5]. Recent reports demonstrate WES’ utility in both clinical [6–8] as well as basic genetics research [9–11]. With growing demand for WES and drop in costs of next-generation sequencing (NGS), WES as a technique requires greater understanding of how experimental design can improve data interpretation and thereby biological outcomes.

Inherent within WES and NGS, however, is much heterogeneity and bias in mean number of times a targeted nucleotide base is sequenced [12]. This heterogeneity in sequencing depth arises due to numerous factors, such as target-enrichment kit used [13, 14], target sequence GC bias [15], PCR amplification bias [16], repeats and pseudo-genes [13], and other experimental design variables. These factors directly and systematically influence sensitivity of WES [14, 17, 18]. While evolving versions of exome-enrichment kits continue to address these biases, effects of technical replicate experimental design in pedigree-based WES are poorly understood.

Pedigree-based WES approaches facilitate the discovery of not only *de novo* variants [19–21], but also multiple-inherited variants [22, 23]. Overall, clinical exome sequencing studies report significantly higher molecular diagnostic yield for pedigree-based approaches compared to single-proband sequencing [21] stressing the importance of understanding WES’s performance in multiple-generation families.

Here, we present an investigation on estimating the proportion of WES target sequence coverage biases that can be eliminated by repetition of the procedure in all individuals in a multiple-generation family. Specifically, we directly compare independent exome-capture libraries generating 18 technical replicates in 6 members of a multiple-generation family (3 per individual). In this study, we evaluated variability and interpreted targeted sequencing within targeted exome regions. Overall, our work reveals the advantages of technical replicate pedigree-based WES in multiple generations, specifically in relation to interpretation of WES derived genotypes [7, 24, 25].

## Results

### Direct comparisons of exome-capture samples in triplicate

**Fig. 1:**
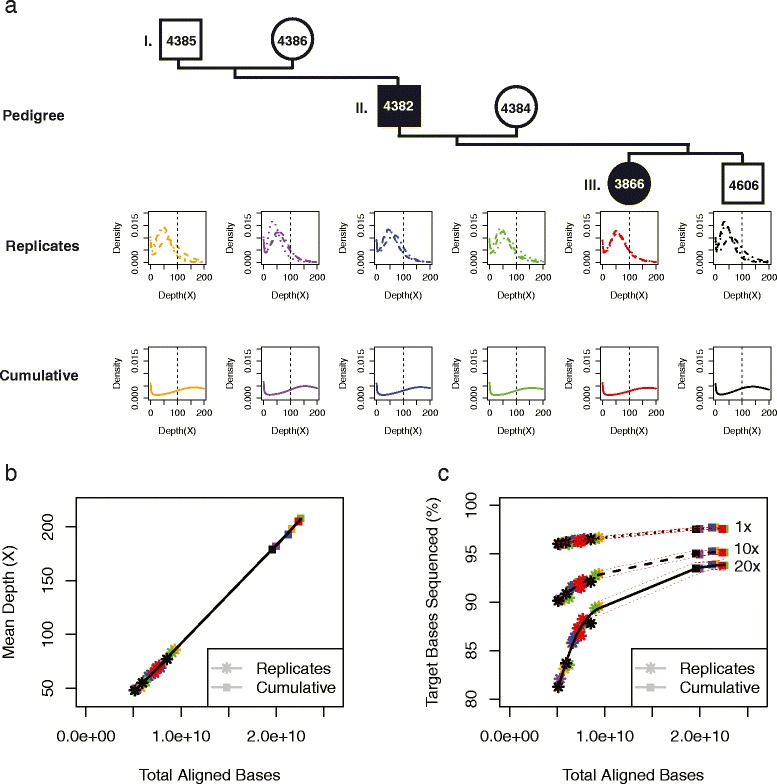
Independent technical replicate target exome capture and aggregate data alignment results for a six member multiple generation family. **a** Probability density function plots of three independent library targeted exome capture experiments as a function of depth of sequencing per targeted base (X) categorized by each member of the six member 3 generation family. **b** Mean depth of coverage as a function of total bases aligned in targeted exome region (62 Mb) for all 18 technical replicates, and aggregate data for 6 individuals derived by merging 3 technical replicate captures per individual. **c** Percent targeted bases sequenced at ≥1x, ≥10x, and ≥20x thresholds as a function of total number of bases aligned in targeted exome region (62 Mb) for technical replicate and aggregate data for each individual. *Black* lines show the predicted *local polynomial regression (loess)* fit to data with default span value of 0.75, and *red* dashed lines represent predicted 95 % confidence interval along the predicted line

We compared the results of independent exome-capture in triplicate for six individuals from three generations (Fig. 1), evaluating them for high-quality bases (>Q20) aligned to a reference human genome (hg19). We compared the alignment results either between technical replicate samples of a single individual or between individuals within the pedigree by utilizing pooled data of all technical replicates for each individual. In total, 18 exome data sets from six individuals were evaluated. Illumina HiSeq2000 sequencing generated 53–98 million paired-end (PE) 100-bp reads per technical replicate library to produce >48x mean alignment target depth of high-quality mapped bases (>Q20) for each technical replicate library (Table 1). This generated 5–9 Gb of high-quality target-aligned data per technical replicate and cumulatively 20–22 Gb (206–235 million PE 100-bp reads) to give 179 - 208x average target coverage for each individual in the pedigree (Additional file 1: Table S1).

**Table 1:** Whole exome sequencing mean target coverage depth and percent target coverage statistics of replicate and aggregate data

WES sample	Mean target depth	% Target ≥1x	% Target ≥10x	% Target ≥20x
ID3866				
Replicate 1	68x	96.2	91.5	86.6
Replicate 2	65x	96.3	91.9	87.3
Replicate 3	71x	96.4	92.3	88.3
Mean ± SE		96.3 ± 0.1	91.9 ± 0.4	87.4 ± 0.8
Aggregate	205x	97.6	95.1	93.8
ID4382				
Replicate 1	69x	96.5	92.1	87.8
Replicate 2	61x	96.3	91.6	85.8
Replicate 3	63x	96.3	91.7	86.4
Mean ± SE		96.4 ± 0.1	91.8 ± 0.4	86.7 ± 1.0
Aggregate	193x	97.7	95.3	93.9
ID4384				
Replicate 1	56x	96.0	90.5	83.5
Replicate 2	68x	96.3	92.0	87.4
Replicate 3	83x	96.5	92.7	89.4
Mean ± SE		96.3 ± 0.3	91.7 ± 1.2	86.8 ± 3.0
Aggregate	208x	97.6	95.1	93.8
ID4385				
Replicate 1	51x	96.2	90.3	83.1
Replicate 2	86x	96.7	92.9	89.6
Replicate 3	61x	96.3	91.6	85.7
Mean ± SE		96.4 ± 0.2	91.6 ± 1.3	86.1 ± 3.2
Aggregate	198x	97.7	95.3	93.9
ID4386				
Replicate 1	64x	96.4	91.6	86.8
Replicate 2	69x	96.3	91.9	87.4
Replicate 3	49x	96.0	90.3	82.1
Mean ± SE		96.2 ± 0.2	91.2 ± 0.8	85.4 ± 2.9
Aggregate	182x	97.6	94.9	93.5
ID4606				
Replicate 1	77x	96.5	92.1	87.8
Replicate 2	48x	96.0	90.1	81.2
Replicate 3	55x	96.2	90.9	83.7
Mean ± SE		96.2 ± 0.2	91.0 ± 1.0	84.2 ± 3.3
Aggregate	179x	97.5	95.0	93.5

Technical replicate data were compared directly for each individual (Fig. 1a). The mean target-depth of sequencing varied linearly with the input of total sequence data and was evident for all technical replicates derived from the six individuals (Fig. 1b; Table 1). The most variable technical replicate depth of sequencing results was from individual ID4385 (51x-86x) and the least variable was from individual ID3866 (69x-71x). Upon aggregation of data from all technical replicates for each individual, the depths of coverage were 179 - 208x for targeted regions (Fig. 1b).

To determine sequencability of targeted bases, we determined the percentage coverage with ≥1x to ≥100x (increments of 10x) in each technical replicate and in each aggregate data set (Additional file 1: Table S2 and Figure S2). This analysis identified minimal variability at ≥1x coverage but appreciable variability at ≥20x coverage (81.2 - 89.6 %) among the technical replicates of each individual (Fig. 1c). Greater variability was observed at higher (≥30x to ≥100x) depth of sequencing thresholds (Additional file 1: Figure S2). Subsequently we restricted our analysis to ≥1x, ≥10x and ≥20x thresholds. We observed that this variability was a function of the total number of bases aligned to target regions. Therefore, we used a *local polynomial regression* (loess) package in the R statistical software to estimate variation in percent target region coverage as a function of sequenced bases aligned to target regions. We used this tool to fit data for technical replicate and cumulative percent target region sequenced (span = 0.75). Using this approach, we predicted a polynomial fit to percent target bases sequenced as a function of total bases aligned in targeted regions, and determined the predicted 95 % confidence interval along the fitted line. Results showed higher standard error at ≥20x relative to ≥1x (Fig. 1c). In addition, at ≥1x we noticed that the fitted line approached saturation as a function of total bases aligned to target. Taken together, this suggested that lower thresholds (≥1x) had lower variability, and ≥20x threshold was highly sensitive to changes in total bases aligned to target (Fig. 1c, especially when aligned data were below 10 Gb). Given these observations, we investigated whether higher depth of sequencing would stabilize this effect at ≥20x threshold and repeated this analysis using the aggregate data for each individual. In addition to sequencing 93.4 - 93.9 % of targeted bases at ≥20x, we observed less influence of input sequence data on the variability of percent target bases sequenced (see predicted 95 % confidence interval at higher depths). Overall, our results supported the conclusion that current exome sequencing results (mean depth of <100x, 10 Gb aligned data) have high variability at the ≥20x coverage threshold.

**Fig. 2:**
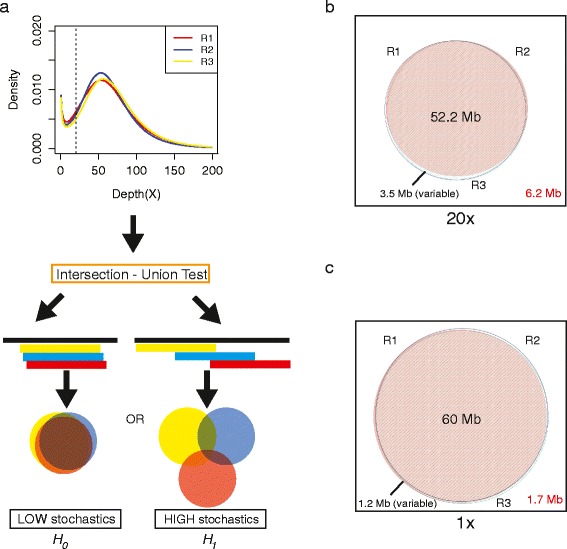
Estimation of stochastic variability between technical replicate targeted sequencing experiments within the same individual. **a** Schematic representation of intersection–union test (IUT) on technical replicate data generated independently in triplicate (R1, R2 and R3). The probability density function was generated from technical replicate data of a single individual (ID3866) with least variable input sequence data. The IUT is performed at preset thresholds to test for low stochastic variability (*H*_*0*_) or the alternative hypothesis of high stochastic variability (*H*_*1*_) (**b**) Area-proportional Euler Venn diagram (eulerAPE v3.0) of targeted bases sequenced in three technical replicates R1, R2 and R3 at ≥20x. The square represents the total targeted bases, and area in *white* as the total number of targeted bases not sequenced at a given threshold (≥20x). **c** Area-proportional Euler Venn diagram (eulerAPE v3.0) of targeted bases sequenced in three technical replicates R1, R2 and R3 at ≥1x. The square represents the total targeted bases (*not proportional* relative to the circles), and area in *white* as the total number of targeted bases not sequenced at a given threshold (≥1x)

### Stochastics in capture and sequencing can be estimated by replicate libraries

**Fig. 3:**
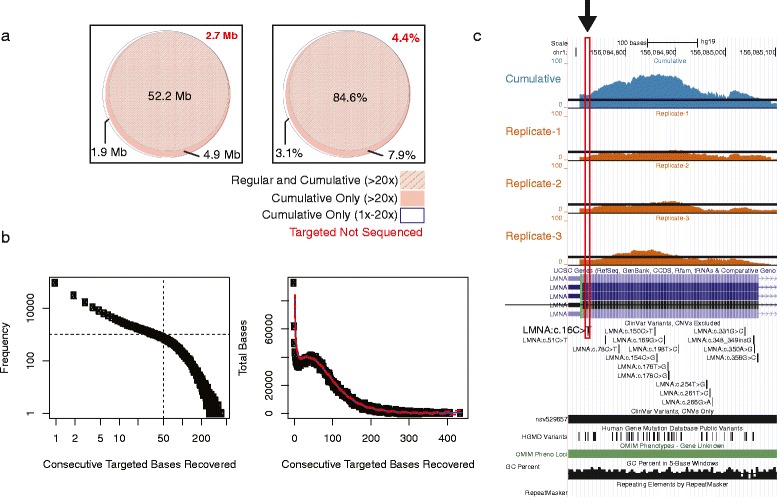
Impact of deep sequencing as estimated by aggregate exome sequence data from replicates in least variable individual. **a** Area-proportional Euler Venn diagram (eulerAPE v3.0) of targeted bases sequenced by standard exome sequencing (regular) and aggregate exome sequencing. Sequenced data are represented within circles of Venn diagram (*black numbers*), whereas targeted and missed by exome sequencing is represented by the square (*red numbers*). *Left panel* represents targeted bases in megabases (Mb), and *right panel* represents the results as percentage of total targeted bases. **b** Distribution analysis of number of consecutive targeted bases recovered by deep sequencing to ≥20x. *Left panel* is a log-log plot of frequency of consecutive targeted bases recovered. *Right panel* plots the distribution of total number of bases sequenced as a function of consecutive targeted bases recovered by deep sequencing to ≥20x. *Red dashed* lines represent 95 % confidence interval of loess predicted to the data (*blue line*). **c** UCSC genome browser screen shot example of *LMNA* exon that illustrates the variability of ≥20x sequencing along the length of the exon. The *black arrow* and *red box* highlight a known disease causing mutation (c.16C > T; p.Q6X) that is consistently missed at the ≥20x threshold by all three technical replicates, but addressed by aggregate sequencing. Aggregate data covers the entire exon to ≥20x

Data from exome sequencing are typically reported as percent targeted bases sequenced at a given sequencing depth threshold. Although informative for the performance of targeted sequencing as a whole, this masks the ‘true’ stochastic nature of per-target-base coverage. In other words, it does not clarify whether a given targeted base achieves the required minimum depth of sequencing if the capture experiment were to be repeated independently. To address this, we analyzed the technical replicate data regarding what fraction of total targeted nucleotides were subject to stochastic genotypeability and sequencing given comparable, equal input sequence data (Fig. 2a). To investigate the relative stochastic variation in coverage at a per-target-base level, we grouped the technical replicate samples by individual. Technical replicate data for all individuals are shown in Additional file 1: Table S1. As proof of principle, we picked the set with the least variable sequence input data (ID3866). The null expectation (*H*_*0*_) was that there would be no appreciable difference between the intersection and the union of technical replicate sets (Fig. 3a). We observed that 52,212,644 bases (84.3 %) of 61,884,224 targeted bases were sequenced at ≥20x coverage in all three technical replicates (intersection), whereas 3,443,727 (5.5 %; P-value = 0.0007; two-independent proportion test) targeted bases were sequenced at ≥20x coverage in at least one but not all three technical replicates (union) (Fig. 2b). Similar variation was observed for the three technical replicates of each of the five other individuals (data not shown).

Because the above results for coverage at ≥20x were theoretically dependent on sequencing input quantity, we repeated the analysis at near ‘predicted’ saturation of capture and sequencability (≥1x coverage). Only 1.9 % of targeted bases were variable among the technical replicates. Specifically, 58,994,725 bases (95 %) were sequenced at ≥1x coverage, whereas 1,203,131 bases (1.9 %) were sequenced in at least one but not all three technical replicates (not significant) (Fig. 2c). This suggested that stochastic variability among these technical replicates contributes little to overall sequencability (≥1x) although it had an appreciable affect on usable (≥20x coverage) sequence data.

### Cumulative technical replicate sequencing improves targeted sequence interpretation

Of the variability within WES target capture regions, 2-3 % arose within protein-coding regions at ≥20x depth of sequencing threshold [26]. To understand whether or not deep sequencing addresses stochastic variability and benefits achieve theoretical maximum coverage (>95 % of targeted bases), we merged the three technical replicate bam files from each subject to generate a single bam file (Additional file 1: Table S1). For each aggregate data set, 20–22 Gb high quality reads covered targeted WES regions; each targeted base had an average of 179 - 208x coverage. Compared to 81.2 - 89.6 % (50.2-55.4 Mb of 61.7 M) coverage of targeted bases at ≥20x in the individual technical replicates, the merged data covered 93.5 - 93.9 % of targeted bases (57.7 – 58.0 of 61.7 M) at ≥20x, suggesting a 4.3 – 12.7 % improvement in coverage (Fig. 3a, Table 1). Each individual’s aggregate dataset recovered 3.4 – 6.4 million bases of variable targeted region.

The distribution of consecutive targeted bases recovered to ≥20x sequencing depth followed a power-law distribution (Fig. 3b). Aggregate data recovered 117,913-170,667 singleton target-base positions (Fig. 3b); the average-size of consecutive bases recovered was ~50 bp (Fig. 3b). In each individual, we identified 17,132 - 40,726 segments greater than 50 bp. We then intersected regions greater than 50 bp with UCSC known-gene protein-coding exons. Intersecting UCSC known-gene coding bases with recovered regions revealed that 8,156 - 22,868 regions (0.5 – 2 Mb) overlapped protein-coding regions, including 12–16 of the 56 genes that the American College of Medical Genetics (ACMG) recommended for return of incidental findings in clinical sequencing [25]. Figure 3c illustrates that current depths of sequencing consistently fail to meet 20x coverage at clinically important sites. For example c.16C > T variant (p.Q6X; chr1: 156,084,725; hg19) in *LMNA*, a cause of autosomal dominant Emery-Dreifuss muscular dystrophy (EMD) [27]. This illustrates how aggregate deep sequencing may help recover variable regions to 20x or greater depth of coverage. Taken together, this analysis not only revealed the advantage of technical replicate sequencing to determine exact targeted regions affected by stochastics under current exome sequencing standards but also demonstrated the utility of merging the technical replicate data to permit interpretation of regions with coverage that is otherwise too shallow.

### Genotyping sensitivity and accuracy to detect *de novo* variants improves with cumulative replicate sequencing

We investigated the effect of stochastic variation on genotyping of variants among technical replicate data sets of the same individual. We included all 18 technical replicates for this analysis. Since depth of sequencing and the relative proportion of representation of the alternate allele play a key role in genotype calling [28], we delineated genotype discordances among technical replicates at varying depths of sequencing. To address this question systematically, we binned each targeted position into 10-19x, 20-29x, and ≥30x bins. We evaluated sites within each of these bins where genotype calls disagreed between technical replicates of the same individual. In total we evaluated 19,424,806, 11,888,469, and 75,145,195 positions in the 10-19x, 20-29x, and ≥30x bins, respectively, and found 65 differences. 62 of these differences were in the 10-19x bin (between technical replicate genotype discordance: 3.2x10^−6^), 3 in the 20-29x bin (between technical replicate genotype discordance: 2.5x10^−6^) and none in the ≥30x bin.

To evaluate the accuracy of WES genotyping at the 65 genotype-discordant sites, we performed Sanger dideoxy chain-termination sequencing. Of the 54 differences for which we could design functional primers (Additional File 1: Table S3), Sanger sequencing showed that 20 (37 %) were heterozygous; 29 (54 %) were homozygous reference, and 5 (9 %) were homozygous non-reference. This demonstrated that 20 heterozygotes were not called in at least one technical replicate in an individual (disagreement rate: 0.64x10^−6^; 20 in 31,313,275 sites tested), while 29 variant sites were falsely called as heterozygotes in at least one technical replicate. The sequence surrounding 13 of the 29 sites mapped to multiple regions of the human reference sequence suggesting that the differences arose from mis-mapping (Additional file 1: Table S3).

Next, we called *de novo* variants that arose in second and third generations of the family. In technical replicate data, for each trio, we found 3–11 (mean: 6.2 ± 3) and 1–8 (mean: 3.3 ± 2) *de novos* at 10x and 20x thresholds, respectively. In aggregated data, we found 4–7 (mean: 6 ± 1.7) and 3–4 (mean: 4 ± 2.3) *de novos* at 10x and 20x thresholds, respectively. Of the 30 sites for which we could design effective primers, Sanger sequencing showed that 4 were true *de novo* variants in technical replicates (Additional file 1: Table S4). Two of the 6 *de novo* variants identified in the aggregate data were missed in technical replicate data. The technical replicate and aggregate variants that did not validate by Sanger sequencing were in regions with problematic GC-content or mappability (Additional file 1: Figure S5 and Table S5) [29]. Taken together, technical replicate data along with aggregate data for a given individual improved the interpretation of NGS genotype calls compared to current WES standards.

## Discussion

In this study, we investigated the utility of independent generation of exome capture libraries for the purpose of interlibrary comparison, the effects of stochastics on targeted genomic region capture, the advantages of aggregate data on targeted resequencing, and finally the effect of the overall process on genotyping and *de novo* detection.

We showed that current accepted exome-sequencing threshold of ≥ 20x is unsaturated at average 50x-100x WES target coverage, since interpretation of the targeted bases varied significantly among technical replicates at a ≥ 20x threshold and not significantly at ≥ 1x threshold. Our results are consistent with current understanding that capture sequencing data interpretation is heavily dependent on amount of exome data generated [14, 22, 28]. Certain targeted regions had variable coverage only as a consequence of input data since aggregate data met the genotyping threshold of ≥ 20x coverage. Controlled analysis done by limiting the effect of input sequence data on overall measurement of stochastics showed variability between technical replicates at the genotypeability threshold of ≥ 20x and this is addressed by aggregating data to higher depths. A recent report by Redin and colleagues [9] support our conclusion that aggregate data is beneficial to the overall interpretation of exome data. They showed that un-interpretable regions within targeted portions can be as low as 3.9 kb (1.8 kb protein-coding) given deep-sequencing (mean coverage: >350x) compared to our observation that it (mean coverage: 40-50x) can be as high as 0.5-2 Mb. Therefore deeper exome sequencing may have potential to improve diagnostic yield for unselected patients, which for rare disorders is currently 25 % (95 % CI: 20–31) in clinical laboratories [30].

Our approach is unique and novel because it addresses the potential library generation-specific sequencing biases that may propagate through the sequencing process and when evaluated, appear as true single nucleotide variants. Our study design comparing technical replicate library data from the same individual provides an added advantage to detect genotyping anomalies that would otherwise be undetectable. This approach however raises the question of whether funding would be better spent on two additional technical replicates rather than on single library preparation and generating additional sequence data. At the time when this study was designed and performed, it cost $300 per replicate library preparation (Illumina TruSeq kit; see Additional file 1: Table S6). However, due to the rapid drop in prices and technological advancements, cost estimates for replicate library preparations currently are around $124 per replicate library (Illumina Nextera; see Additional file 1: Table S6). Using the triplicate library approach would therefore equate to an extra cost of $248 per individual sequenced, when compared to single library preparation coupled with deep sequencing approach (same sequence data output). Estimating current sequencing costs at $0.1 per million bases sequenced (http://www.genome.gov), we assess that $248 would theoretically allow for purchase of 2.48 Gb of additional sequence data per sample. Given 70-85 % of 2.48 Gb would pass post-alignment and quality-filter analysis, 1.7-2.1 Gb mappable data would be available for interpretation. Given our observations of target-base coverage saturation at ≥ 20x threshold for 20–23 Gb aggregate user-quality data, we conclude that an extra 1.7-2.1 Gb per sample would minimally alter the overall interpretation of targeted regions under evaluation (see Additional file 1: Figure S6).

Finally, we argue that our approach and findings are consistent with other studies that note benefits of replicate exome comparisons for variant detection and replicated exome merging for variant calling accuracy [31]. Benefits of this approach may also minimize the stochastic branching process of allele-distribution in exome datasets derived from a single library generation process and may additionally help mitigate library specific amplification biases [18].

## Conclusions

We describe a method to evaluate the reproducibility and stochastics in exome library preparation, and delineate the advantages of aggregating the data derived from technical replicates. The implications of this study are directly applicable and provide an opportunity to rapidly, efficiently, and accurately diagnose patients.

## Methods

### Patients

Patients accepted into the NIH Undiagnosed Diseases Program (UDP) were enrolled in clinical protocol 76-HG-0238 approved by the Institutional Review Board (IRB) of the National Human Genome Research Institute. The individuals or their guardians gave written, informed consent.

### Genomic DNA extraction

Genomic DNA was extracted from peripheral whole blood using the Gentra Puregene Blood kit (Qiagen, Inc.), which employs modified salting-out precipitation according to the manufacturer’s protocol as previously described [2]. DNA was eluted in 250 – 1000 μL at a concentration of up to 35 μg/mL.

### Independent exome-library preparation and capture

Independent exome-libraries were generated in triplicate (3 technical replicates) for each subject (Fig. 1b) using the Illumina TruSeq DNA Sample Preparation kit (version 2) according to the manufacturer’s protocol. Pre-enrichment, all independent libraries were pooled and multiplexed up to 6-samples. Pooled libraries were captured in-solution for isolating exonic regions of interest in the human genome using hybrid selection with TruSeq Exome Enrichment kit, version 2 (Illumina, Inc.) as per the manufacturer’s protocol. The kit targeted 62 Mb of the human genome using 95mer probes that selected target libraries of 300–400 bp and enriched 265–465 bases centered symmetrically on the midpoint of the probe.

### Exome sequencing and data processing

Paired-end sequencing was performed on the Illumina HiSeq 2000 instrument generating 100-bp reads. The output reads from the Illumina Genome HiSeq 2000 were mapped to the reference haploid human-genome sequence (Genome Reference Consortium human genome build 37; human genome 19) with the use of the *eland* (Illumina, Inc) generating per-sequencing-lane bamfiles. Fastq files derived from per-sequencing-lane bamfiles were re-aligned to the reference sequence using the *Novoalign* program (http://www.novocraft.com) with default parameters; data for this stage were grouped by technical replicate samples. *SAMtools* [32] was used to identify and remove PCR duplicates. Each exome sequencing result was either saved as a technical replicate bamfile or a merged bamfile (aggregate data) derived by merging all three technical replicates of a given individual. Genotypes were called at all positions where there were high-quality sequence bases (Phred-like Q20 or greater) using a Bayesian algorithm (Most Probable Genotype – MPG) [33]. Nucleotide coverage queries and read depth analyses were performed using *SAMtools* [32]. The depth of coverage was calculated from bamfiles with *SAMtools* and custom PERL scripts. Descriptive statistics for sequencing data analysis were obtained using R statistical software (version 3.1.2).

### *De novo* mutation analysis

For *de novo* mutation (DNM) detection analysis, all family members’ pedigree information was formatted into trio data. Each individual’s data were organized into technical replicate bamfiles. For each technical replicate bamfile, genotypes were called in targeted regions and these data were binned by target region coverage: 10-19x, 20-29x, and ≥30x bins. Variants were classified as DNMs in scenarios that did not fit Mendelian inheritance patterns for each trio, similar to methodologies established in previous publications [34, 35]. We iterated this procedure for all possible trios and all bins. Technical replicate DNM concordance was computed using PERL scripts.

### Bioinformatics data analysis

The data were chiefly analyzed and annotated by means of a bioinformatics pipeline that was developed in-house, mainly consisting of PERL scripts. Data were formatted for parallel computational processing using GNU Bash scripting (http://www.gnu.org/software/bash) on a Linux operating system (http://linux.org). Parallel and batch compute job tasks were submitted, queued, and managed by Portable Batch System (PBS) computer software in a Linux cluster environment. Job tasks were processed on National Institutes of Health (NIH) Biowulf system via login node (biowulf.nih.gov). All nodes within this system are connected to a 1 Gb/s switched Ethernet network, while sub-sets of nodes are on high-performance Infinipath or Infiniband networks (16 Gb/s bandwidth with very low latency). Job tasks were mostly processed on 8 x 2.67 GHz Intel X5550, 32 x 72 GB nodes (352 nodes) in this system. Data were accessed from RAID-6 file systems mounted over Network File System (NFS; Sun Microsystems) or General Parallel File System (GPFS; IBM).

### Statistical analysis

R statistical software (version 3.1.2) (www.r-project.org) was used for statistical data analysis and plotting figures. Non-parametric regression method, Local Polynomial Regression Fitting (*loess*) was used for local polynomial fitting of percentage of exome target region sequenced data (span = 0.75). Fitting was locally controlled by alpha parameter (span (α)), which determined the size of the regression neighborhood. To test for stochastics in target bases sequenced, we used two independent proportion test (*prop.test*)*.* This tested the null that the probabilities of success (coverage of same sequence bases) in several groups are the same (conf.level = 0.95).

### Sanger sequencing

For Sanger sequencing verification of genotypes called by NGS, oligo-nucleotide primers were synthesized by Integrated DNA Technologies. The regions containing the suspected single nucleotide variants (SNVs) were amplified by polymerase chain reaction (PCR) using 50 ng of genomic DNA derived from patient peripheral blood, the listed primers and Qiagen HotStar Taq Plus under the following conditions: 95 °C x 5 min denaturation followed by 40 cycles of 95 °C x 30 s, 55 °C x 30 s, 72 °C x 30 s. Residual primers and nucleotides were removed with ExoSAP-IT reagent (USB, Cleveland, OH, USA). The amplicons were then sequenced using BigDye**®** terminator chemistry by Macrogen (Rockville, MD) and compared to Human Genome reference sequence (GRCh37; assembly hg19) using Sequencher (GeneCodes, Ann Arbor, MI, USA).

## Additional files

Additional file 1: Figure S1. Systematic approach to study exome capture variability in exome-sequencing (A) Three-generation pedigree in which two individuals have an undiagnosed disease that segregated as an autosomal dominant disorder and a *de novo* variation arose in the second generation. (B) Model of individual subject sample blood DNA processing and sequencing. A sample of blood went through DNA isolation, and independent libraries (in triplicate) were sequenced to appropriate comparable depth and analyzed for various quality control parameters, target coverage, read depth and nucleotide variation detection. (C) Schematic illustration of sequencing read depth vs. targeted genomic region in relation to exome sequencing in replicate. Listed are also the main approaches taken in this study to analyzed exome replicate data. (D) Two main hypotheses tested using replicate exome data: (i) Biases in sequence capture resulting in poor coverage are addressable through repetition (ii) Library replication is beneficial to overall interpretation of sequence variation data. **Figure S2.** Titration of percentage targeted exome sequenced as a function of depth of sequencing thresholds in all three replicates per sample. Error bars show standard error for replicate sequencing. As expected, higher depth of sequencing thresholds (x-axis) result in higher-coverage (y-axis) variability in replicate exome data. **Table S2.**Titration of percentage target exome sequenced as a function of depth of sequencing thresholds (attached excel file). **Table S3.** Primers used for and results of Sanger sequencing analysis for resolution of replicate discordances in NGS data. **Table S4.** Primers used for and results of Sanger sequencing validation of *de novo* variants detected using NGS. (Concordant NGS and Sanger genotypes are highlighted in yellow). **Figure S3.** Box-plot of GC-content distribution in all first-exons (*blue*) and high-GC content (>70% GC; >=50 bp length). **Table S5.** Evaluation of coverage of targeted exons with high GC content (attached excel file). **Table S6.** Quote from Illumina for exome enrichment kits. Quotes in red indicate costs when the study was undertaken. *Nextera* prices, and other kit prices (in *white*) reflect current costs per sample (see last column). (DOC 1 mb) Additional file 2: Table S2. Titration of percentage target exome sequenced as a function of depth of sequencing thresholds. (XLSX 59 kb) Additional file 3: Table S5. Evaluation of coverage of targeted exons with high GC content. (XLSX 56 kb)
