# Supplementary material for: Replicate exome-sequencing in a multiple-generation family: improved interpretation of next-generation sequencing data
Source: BMC Genomics. 2015 Nov 25;16:998. doi: 10.1186/s12864-015-2107-y (PMC4659195; doi:10.1186/s12864-015-2107-y)
Supplement: Additional file 1: Figure S1. — Systematic approach to study exome capture variability in exome-sequencing (A) Three-generation pedigree in which two individuals have an undiagnosed disease that segregated as an autosomal dominant disorder and a de novo variation arose in the second generation. (B) Model of individual subject sample blood DNA processing and sequencing. A sample of blood went through DNA isolation, and independent libraries (in triplicate) were sequenced to appropriate comparable depth and analyzed for various quality control parameters, target coverage, read depth and nucleotide variation detection. (C) Schematic illustration of sequencing read depth vs. targeted genomic region in relation to exome sequencing in replicate. Listed are also the main approaches taken in this study to analyzed exome replicate data. (D) Two main hypotheses tested using replicate exome data: (i) Biases in sequence capture resulting in poor coverage are addressable through repetition (ii) Library replication is beneficial to overall interpretation of sequence variation data. Figure S2. Titration of percentage targeted exome sequenced as a function of depth of sequencing thresholds in all three replicates per sample. Error bars show standard error for replicate sequencing. As expected, higher depth of sequencing thresholds (x-axis) result in higher-coverage (y-axis) variability in replicate exome data. Table S2.Titration of percentage target exome sequenced as a function of depth of sequencing thresholds (attached excel file). Table S3. Primers used for and results of Sanger sequencing analysis for resolution of replicate discordances in NGS data. Table S4. Primers used for and results of Sanger sequencing validation of de novo variants detected using NGS. (Concordant NGS and Sanger genotypes are highlighted in yellow). Figure S3. Box-plot of GC-content distribution in all first-exons (blue) and high-GC content (>70% GC; >=50 bp length). Table S5. Evaluation of coverage of targeted exons with high GC cont [file 12864_2015_2107_MOESM1_ESM.doc]

**Additional file 1**

# Replicate exome-sequencing in a multiple-generation family: improved interpretation of next-generation sequencing data

### Praveen F Cherukuri^1, 4, §^, Valerie Maduro^1^, Karin V Fuentes Fajardo^1^, Kevin Lam^1^, NISC Comparative Sequencing Program^2^, David R Adams^1, 3^, Cynthia J Tifft^1, 3^, James C Mullikin^2^, William A Gahl^1, 3^, Cornelius F Boerkoel^1^

^1^ NIH Undiagnosed Diseases Program, Common Fund, Office of the Director, NIH, Bethesda, Maryland, USA

^2^ NIH Intramural Sequencing Center, National Human Genome Research Institute, NIH, Bethesda, Maryland, USA

^3^ Office of the Clinical Director, National Human Genome Research Institute, NIH, Bethesda, Maryland, USA

^4^ Inova Translational Medicine Institute, Inova Health System, Falls Church, Virginia, USA

**A.**

**B.**

**
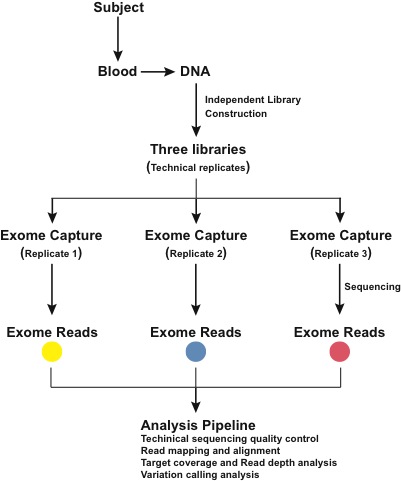
**

**C.**

**
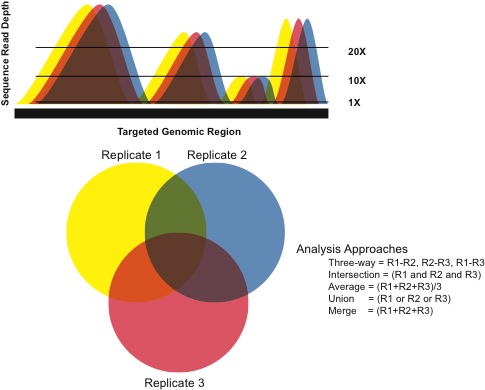
**

**D.**

**
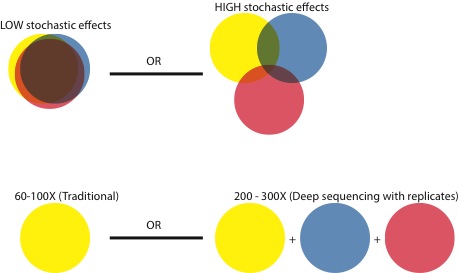
**

**Figure S1.** Systematic approach to study exome capture variability in exome-sequencing (A) Three-generation pedigree in which two individuals have an undiagnosed disease that segregated as an autosomal dominant disorder and a *de novo* variation arose in the second generation. (B) Model of individual subject sample blood DNA processing and sequencing. A sample of blood went through DNA isolation, and independent libraries (in triplicate) were sequenced to appropriate comparable depth and analyzed for various quality control parameters, target coverage, read depth and nucleotide variation detection. (C) Schematic illustration of sequencing read depth vs. targeted genomic region in relation to exome sequencing in replicate. Listed are also the main approaches taken in this study to analyzed exome replicate data. (D) Two main hypotheses tested using replicate exome data: (i) Biases in sequence capture resulting in poor coverage are addressable through repetition (ii) Library replication is beneficial to overall interpretation of sequence variation data.

**Table S1.** Whole-exome sequencing statistics

| **Subject** | **WES replicates** | **Reads** | **Reads per subject** | **HQ aligned bases (>Q20)** | **Aligned data per subject (Gb)** | **Mean target depth** | **Cumulative mean target depth** |
| --- | --- | --- | --- | --- | --- | --- | --- |
| ID4382 | R1 | 81,634,553 | 223,093,378 | 7,820,142,587 | 21 | 69x | 193x |
|  | R2 | 69,474,161 |  | 6,611,056,076 |  | 61x |  |
|  | R3 | 71,984,664 |  | 6,830,770,354 |  | 63x |  |
| ID4384 | R1 | 65,600,677 | 237,676,033 | 6,218,410,648 | 23 | 56x | 208x |
|  | R2 | 77,194,648 |  | 7,335,255,891 |  | 68x |  |
|  | R3 | 94,880,708 |  | 9,013,332,569 |  | 83x |  |
| ID4385 | R1 | 60,208,741 | 227,289,554 | 5,757,799,387 | 22 | 51x | 198x |
|  | R2 | 98,184,866 |  | 9,338,565,130 |  | 86x |  |
|  | R3 | 68,895,947 |  | 6,537,399,925 |  | 61x |  |
| ID4386 | R1 | 75,187,099 | 209,566,961 | 7,207,028,322 | 20 | 64x | 182x |
|  | R2 | 78,574,351 |  | 7,472,083,715 |  | 69x |  |
|  | R3 | 55,805,511 |  | 5,294,736,202 |  | 49x |  |
| ID4606 | R1 | 89,878,496 | 206,349,984 | 8,509,675,353 | 20 | 77x | 179x |
|  | R2 | 53,789,895 |  | 5,124,375,909 |  | 48x |  |
|  | R3 | 62,681,593 |  | 5,940,048,539 |  | 55x |  |
| ID3866 | R1 | 78,730,369 | 235,079,723 | 7,466,896,309 | 22 | 68x | 205x |
|  | R2 | 75,288,996 |  | 7,169,824,776 |  | 65x |  |
|  | R3 | 81,060,358 |  | 7,695,671,462 |  | 71x |  |


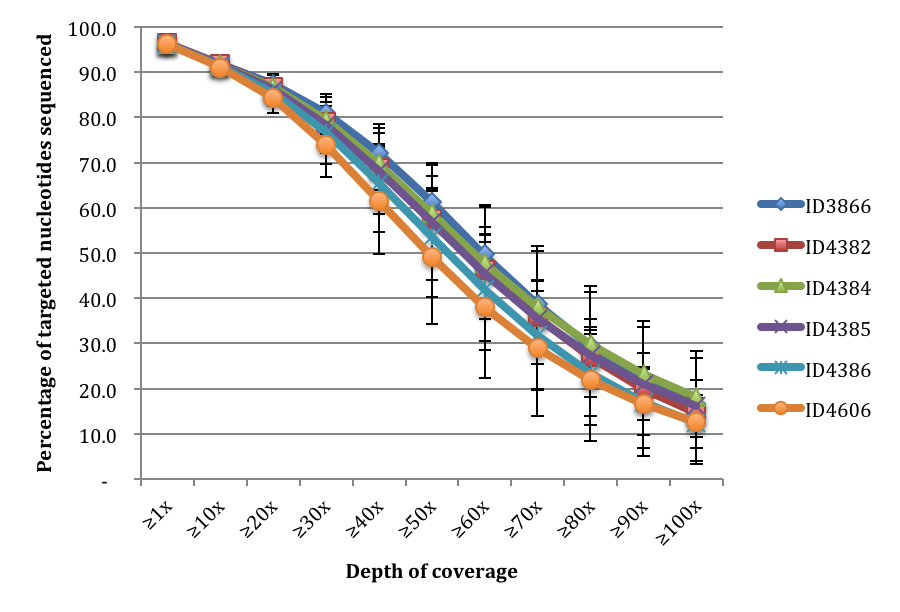


**Figure S2.** Titration of percentage targeted exome sequenced as a function of depth of sequencing thresholds in all three replicates per sample. Error bars show standard error for replicate sequencing. As expected, higher depth of sequencing thresholds (x-axis) result in higher-coverage (y-axis) variability in replicate exome data.

**Table S2.** Titration of percentage target exome sequenced as a function of depth of sequencing thresholds (attached excel file).

**Table S3.** Primers used for and results of Sanger sequencing analysis for resolution of replicate discordances in NGS data.

| ID | Chr | Position  (hg19) | Ref | NGS Bin | Sanger Genotype | Homozygous/ Heterozygous | Reference/ Variant | Forward Primer Sequence | Reverse Primer Sequence |
| --- | --- | --- | --- | --- | --- | --- | --- | --- | --- |
| ID4382 | chr10 | 42399305 | T | 10x-19x | TT | Homo | Ref | GTTTTCAGGCCGATGGTAGA | GAAGGCCTCAAAGAGGTCAA |
| ID4382 | chr11 | 62375043 | T | 10x-19x | TT | Homo | Ref | GGGTGATAGAGCAAGGCACT | CTTCAACCCTCGTGACAGC |
| ID4382 | chr11 | 86970396 | A | 10x-19x | AG | Hetero | Var | CCATAGTCTCTCCGACCAGG | CAATGATCCCATTCACTGAAACT |
| ID4382 | chr11 | 123455109 | G | 10x-19x | NA |  |  | CCTTTCTGGAACTGCCTTGA | ACTCCCTCCCTGACTGGTCT |
| ID4382 | chr15 | 95019835 | A | 10x-19x | AA | Homo | Ref | CTGCTGGGATTGTAATGGGA | GCTGGCCAGATTTCAGGTT |
| ID4382 | chr16 | 46400108 | C | 10x-19x | CC | Homo | Ref | AGGTGATTTCATTCAAGTCCA | GAATCGAGTGGAATCATCGAA |
| ID4382 | chr16 | 89858184 | A | 10x-19x | AA | Homo | Ref | TAGTCACAGCAAGGCAAGGG | GTTGGCCATTTGCAAGAAGT |
| ID4382 | chr16 | 90241210 | G | 10x-19x | CC | Homo | Ref | CAGCAATGACATCATCACCC | CAGGGGCTAGCAGGAGTGTA |
| ID4382 | chr17 | 25290634 | T | 10x-19x | GG | Homo | Var | AGAAAAGAAGGAGGGCGGT | GTGCAAGGATAAGAAGCCCT |
| ID4382 | chr18 | 7031889 | C | 10x-19x | CC | Homo | Ref | GCCTGGGTGACAAGGTAAGA | TTGGAGGAATTTGTCAACCC |
| ID4382 | chr2 | 3481637 | T | 10x-19x | CC | Homo | Var | CTGAAGTTTTCCTCTGCCTGA | ACACAGAACACCAGAGCACG |
| ID4382 | chr2 | 131985793 | C | 10x-19x | CC | Homo | Ref | GTTTCTTGCCCATCAAAGGA | TCACCTGATATGTGAGATGCAA |
| ID4382 | chr2 | 133015766 | A | 10x-19x | GG | Homo | Var | GTCGGAGGCAGAACGGTAG | GCTCCCGGCCTACTTATCTA |
| ID4382 | chr4 | 49141699 | G | 10x-19x | NA |  |  | TCCATTCCTTTCCACTCGG | AATGGTATGGAATCAACCCG |
| ID4382 | chr4 | 49156543 | C | 10x-19x | NA |  |  | TCGGGTTGATGCCATTCTAT | AACCCGAGTGGAATGGAG |
| ID4382 | chr4 | 49560387 | A | 10x-19x | CT | Hetero | Var | TCCATTACTCTGCCAGCACTT | AGGAGAAAGTGAGGTGGCCT |
| ID4382 | chr4 | 147560411 | T | 10x-19x | GG | Homo | Var | GTGAGCTCAACTTCGCACAG | CAGATAAAGGGGGCTGTCAA |
| ID4385 | chr10 | 42392667 | C | 10x-19x | CC | Homo | Ref | GGAATCGAATGGAAACACCA | TCGATTCCACTCAATGATTCC |
| ID4385 | chr10 | 135455555 | C | 10x-19x | CC | Homo | Ref | GGAAATCCTATGGAATCTCCTTTT | CAACTTTCACTTTATCTCCATGC |
| ID4385 | chr12 | 2870720 | A | 10x-19x | AG | Hetero | Var | ATGAATGATGACAGGGAGGC | CTCAGGCCTAGATTTGCCAC |
| ID4385 | chr14 | 107109691 | C | 10x-19x | NA |  |  | GTGAGGAGTCCACCTTCCAA | TTTTAAAGAACTGCTTACCTTTCC |
| ID4385 | chr14 | 107109692 | T | 10x-19x | NA |  |  | GTGAGGAGTCCACCTTCCAA | TTTTAAAGAACTGCTTACCTTTCC |
| ID4385 | chr16 | 46388483 | C | 10x-19x | CC | Homo | Ref | TTCCTTTCGATCATTCCCTT | TCATCGAATGGGATCAAATG |
| ID4385 | chr17 | 10547486 | C | 10x-19x | CC | Homo | Ref | AGGCGGATGGATCACTTG | CCAGCAACTGGATACGAAGC |
| ID4385 | chr19 | 18477940 | C | 10x-19x | CC | Homo | Ref | ATCCTCATGTGGGCTAGGTG | ACACCCCTCAGCCTGTCTT |
| ID4385 | chr1 | 17008613 | C | 10x-19x | CT | Hetero | Var | GCCACAACTCATCCTCATCA | TTGTCCTCATGACCAAACCT |
| ID4385 | chr1 | 213003372 | A | 10x-19x | AT | Hetero | Var | TAAGGAGAAGATTGCCTGGG | GCCATATTTGGATAAAGTGCATC |
| ID4385 | chr20 | 31518552 | C | 10x-19x | CG | Hetero | Var | TGTGGACCTAGACAATGGGG | GGATTAGCAGGCAGGAAGG |
| ID4385 | chr20 | 31518553 | G | 10x-19x | AG | Hetero | Var | TGTGGACCTAGACAATGGGG | GGATTAGCAGGCAGGAAGG |
| ID4385 | chr2 | 92315361 | T | 10x-19x | NA |  |  | CCTCCCATAAAAACTAGATAGAAGC | ACGAAATCCTCAAGGCCAG |
| ID4385 | chr2 | 216974289 | G | 10x-19x | AG | Hetero | Var | TTTCAGGCCTAGCAGGAAAC | GCTAGGGAGTGGAATGGGAT |
| ID4385 | chr6 | 32548212 | C | 10x-19x | AG | Hetero | Var | AGCTGGCTTCACCTCTCACT | TCTCAGTATATGAGTGGCCCTG |
| ID4385 | chr6 | 32634139 | C | 10x-19x | NA |  |  | TAAGTCCAGGCAGTCTTGGG | TGCTTTTCCCTTCGTCTCAG |
| ID4385 | chr6 | 38906072 | A | 10x-19x | NA |  |  |  |  |
| ID4385 | chr7 | 23808506 | A | 10x-19x | AG | Hetero | Var | GGTGGAGAGTGGTGGATGTT | TGCTACAAGACGGTGCAGAG |
| ID4385 | chr8 | 17198730 | G | 10x-19x | AG | Hetero | Var | CTTCGCAACAATTGAACTGC | GTGCCTAGAGGACGAGCAGA |
| ID4385 | chr9 | 37746913 | A | 10x-19x | AG | Hetero | Var | GAGAGGCTACCACGACCTGA | CACCCCTGCCTTCTTGAGTA |
| ID4385 | chr9 | 66345248 | G | 10x-19x | GG | Homo | Ref | CGCTTCAAATAACCCATTCG | TACACGGAAGCCGTTGATCT |
| ID4386 | chr10 | 32856445 | A | 10x-19x | NA |  |  | TTTTAAGGAGACTTTTATTCCAGAAG | TTGTCTCAGTTTTCCCCTCAC |
| ID4386 | chr10 | 42398648 | A | 10x-19x | NA |  |  | GATTTCAAGATATTTGAGGCCAA | GAAATCCCATTTCCAACGAA |
| ID4386 | chr10 | 81342100 | T | 10x-19x | CT | Hetero | Var | CTTCTGGGGCTCTAAGGACA | AGTGAAGGCTCTGTGTGCTG |
| ID4386 | chr10 | 102890421 | G | 10x-19x | AG | Hetero | Var | TCCAAAGGCTTCTTGTCTGG | CTCTGCCCAACTGGAGAATC |
| ID4386 | chr10 | 135455784 | G | 10x-19x | GG | Homo | Ref | ACTGCTGCAAAACCTGCTAC | GCTCCCTATGTTTTCCAGCTC |
| ID4386 | chr11 | 67164791 | T | 10x-19x | TT | Homo | Ref | AAGAGATCTCCAGTGGTCGG | GCCTTCACCCTCACTGTCTT |
| ID4386 | chr13 | 111642891 | C | 10x-19x | CT | Hetero | Var | TCGGAGTTGAATGCATTTGT | TGTTTTTGTTCCACAACACTCC |
| ID4386 | chr15 | 102300121 | T | 10x-19x | NA |  |  | TGCTGTCCAACCTGTACTCG | GCCTCCACGAGTGTCTTCTC |
| ID4386 | chr19 | 6902229 | G | 10x-19x | CC | Homo | Var | TTCCAGGGTCTCAAAGCATC | CTCCATCTCAAAACAAAACCA |
| ID4386 | chr20 | 26062170 | G | 10x-19x | GG | Homo | Ref | AAAGTCACTGCCTGGTCCAC | TCTGGGACTGGGTCATGG |
| ID4386 | chr20 | 32161783 | T | 10x-19x | TT | Homo | Ref | CAGGCTTGAGCCACCACA | CCTTGCCAGGACCTGTATTT |
| ID4386 | chr2 | 133014832 | C | 10x-19x | CC | Homo | Ref | CCACAGACAGGAGGGAGGTA | AGGGAAGCTCGTCGCCTACT |
| ID4386 | chr2 | 133062223 | C | 10x-19x | CT | Hetero | Var | AAACAAAACATGGTGTGGGG | CACCCCTACTCCTGGCTACA |
| ID4386 | chr3 | 9788168 | C | 10x-19x | CT | Hetero | Var | ATGGACTGAACCAAACTGGG | AAGCCATCTGAGTCTGGGTG |
| ID4386 | chr4 | 140217010 | G | 10x-19x | CG | Hetero | Var | GAAGGTCACTGCAGGACGAA | CAGCACGACACTTTTCTTTCTT |
| ID4386 | chr5 | 15937663 | C | 10x-19x | CC | Homo | Ref | TTTCCTCATTTCTCATGGGC | AGTCATTGACAGAGCTGGGG |
| ID4386 | chr6 | 111896797 | A | 10x-19x | AA | Homo | Ref | TGGATCACGAGGTGAAGAGA | CTAGGGACCAGCCACATCAC |
| ID4386 | chr7 | 62517883 | T | 10x-19x | TT | Homo | Ref | GCACCTGGCCTGTTAATGTT | GATCGCACAATTTCACTCCA |
| ID4386 | chr7 | 62517895 | T | 10x-19x | TT | Homo | Ref | CTCATAGCAGCAAAGCCTGT | GGTGACACAGTGAGACTCTGC |
| ID4386 | chr7 | 89875044 | G | 10x-19x | AG | Hetero | Var | CGGCATCAGGAACAAGTCTA | GAACATCACTCGGTAACAGGG |
| ID4386 | chr8 | 12426206 | C | 10x-19x | CC | Homo | Ref | AAGGAACCAGGGCTCAGA | AATTAGGGGATGGACGTGTTC |
| ID4386 | chr8 | 107227677 | G | 10x-19x | GG | Homo | Ref | TGTTTCATCCCTTCTCTCCC | CCCCTGAGCTGCTAGGTGTA |
| ID4386 | chr9 | 177528 | C | 10x-19x | CC | Homo | Ref | CGCAGTCGGATTGACATTTA | GTATTTCAAACGTGCCGCTC |
| ID4386 | chr9 | 67293280 | C | 10x-19x | AC | Hetero | Var | TTCACCTTTGGAACTCTGGG | CTTTATACAGTCTCGGCAGCA |
| ID4382 | chr9 | 100777705 | G | 20x-29x | GG | Homo | Ref | TGCTGTGCATTTTGCTTTTC | GTTGGGGTGAAGTCCACAAG |
| ID4385 | chr17 | 39507148 | T | 20x-29x | TT | Homo | Ref | GAACCAGTTGCAGTTGCTCA | CTGCACCCCACTGAAGAAG |
| ID4386 | chr22 | 16364971 | C | 20x-29x | CC | Homo | Ref | GGCAGGTCAGTGCTGCTC | ACTTCAGGGCCAGAGTCAAC |

Color code: blue = agree; red = disagree ; green= both agree, but likely wrong as homozygous on both platforms

Abbreviations: Chr: chromosome, ID: identifier, Ref: reference sequence

**Table S4.** Primers used for and results of Sanger sequencing validation of *de novo* variants detected using NGS. (Concordant NGS and Sanger genotypes are highlighted in yellow).

| Chr | Position (h19) | Ref Allele | NGS Gen. | MPG Score | Depth of Coverage | Sanger Genotype | Forward Primer Sequence | Reverse Primer Sequence |
| --- | --- | --- | --- | --- | --- | --- | --- | --- |
| chr16 | 89922030 | G | AG | 195 | 132 | AG | GTTTACCAGGGAAGCAGCTG | TTCAGTGGAGGCCGTGAG |
| chr22 | 16287339 | C | CT | 37 | 65 | CC | AGTGTCCTTGAGCATGACGA | TTTCATGGAGCCGAGGTACC |
| chr10 | 65225918 | A | AG | 13 | 19 | CC | GTCCAGATCCAGAGGCGG | TTCACTTTCCCCTCACCTCC |
| chr12 | 12503081 | G | GT | 10 | 17 | GG | CATCCAGGTGCGAGGACA | CTAACCACAAAACCCGCCAG |
| chr17 | 18563926 | A | AG | 11 | 15 | GG | AGGGAAGCTGCTTATCCTCA | AGACACCAGGGCATACTTCC |
| chr17 | 62009690 | C | AC | 13 | 19 | CC | GACAGAGGAGGAGGGTGGA | GGTAAGCACAGACAGAGGGG |
| chr1 | 35334609 | G | CG | 12 | 14 | GG | AGGGCCTTGTCTTCTGTGG | CAAGGTTCAAGCGCTCCAAT |
| chr4 | 89013470 | C | AC | 12 | 14 | CC | TGGGTTCTTTCATGTTTAATTCAG | GCAAAATTGTAAACACACAATGAC |
| chr7 | 76103831 | A | AG | 19 | 18 | AA | AAGTGGTCCTCCCACCTTG | GCACTGGCACAAGAATTTGA |
| chr7 | 76103832 | G | GT | 19 | 18 | GG | AAGTGGTCCTCCCACCTTG | GCACTGGCACAAGAATTTGA |
| chr8 | 65492853 | C | AC | 11 | 15 | CC | TTCAGACTTGCGGCCAGATA | GAAGAAGACTCGCCTGTTGG |
| chr5 | 64920139 | T | AT | 242 | 187 | AT | ATCCCACCTATCGAAGCCTG | ATCTGGAGTTTTCACCCCAA |
| chr5 | 141337911 | T | CT | 326 | 200 | CT | CACTTCAGCAAATGATGGACA | CAGGGCAGAGTCAAGACAGA |
| chr7 | 100637074 | G | AG | 97 | 152 | GG | TCAACTCACACAACGCCTTC | TGCTGCTGTAGACGGTGGTA |
| chr1 | 152276616 | G | GT | 16 | 23 | GG | TCTGCATGATGAGTGCCTGA | ATCGTGGATCTGCTCAGGAG |
| chr15 | 82336383 | G | GT | 11 | 15 | GG | ATGGCACTGAGGCTTCTCC | CAGACGCACACGTACATCG |
| chr17 | 13928438 | A | AG | 11 | 12 | AG | GACTGATGCTGGTGACCCTT | GCACCTCCTGCACACAGATA |
| chr19 | 50528787 | T | GT | 19 | 18 | GT | CTGTACTTGGGGACTGGGG | GAAGAGCAAGCGGTAGGTTG |
| chr22 | 24647843 | G | AG | 17 | 21 | GG | CCCGGACACTTGACATCAGT | GCTGTGGTACCTGTGCAATC |
| chr22 | 24647857 | C | CG | 32 | 35 | CC | GGTTACCTGGGCACTCTGTG | CTAGGGTCCTTGTCCTGCTC |
| chr8 | 7718180 | T | CT | 10 | 14 | TT | GATCTGCGTGGGCTTTTTAG | GCCCTTGGGATACTTCAACA |
| chr14 | 102689401 | G | GT | 13 | 13 | GG | AGACTGCTTTGTTTGCAGGG | CATTCAAGGTTGTCTTCTGACC |
| chr14 | 105846449 | A | AT | 12 | 21 | AA | GTCTGACTGGGCTGTGGTGT | AGCTGGAGTGAATTTTGGGA |
| chr17 | 46621100 | C | AC | 10 | 16 | CC | TCCTTCTCCAGTTCCAGCAG | CTGAGGTTGGGCTGAGTACA |
| chr18 | 12793250 | G | GT | 12 | 21 | GG | CTCTTGACCCACCTGGACAT | GGGATAGTCAGGTAAACAGTTGG |
| chr19 | 3611722 | G | GT | 13 | 20 | GG | TTGCATCAGGACCCTAGGC | TCAGGCCGCATCTTTCTTC |
| chr19 | 5244013 | C | AC | 10 | 17 | CC | CACGGTGTAGGTCTCGTCC | ACTACACCATGGAACCGGAG |
| chr1 | 184661932 | A | AG | 22 | 29 | AG | GCCAGCACCAGACTACTACA | GCTTAATGAGACTTCCACCCC |
| chr1 | 231473246 | G | GT | 30 | 24 | GG | CTCTCCAGGCTGCTTTTCTG | GTCTACCAGAACTACCGGCA |
| chr9 | 137716475 | G | GT | 13 | 20 | GG | AGGAGAGGTGCTCCATGCT | CCCTCACCATCTGGGAAGT |

**GC-Content Analysis**

To test if aggregate data improves sequencing of high-GC content regions, we evaluated all first exons of known UCSC genes (hg19; 78,827 total (47,766 – unique set)) that had greater than 70% GC-content and spanning ≥50 bases. We found roughly 1% (4,507) of all first exons (total = 47,766) had GC-content greater than 70% and spanned ≥50 bases. All first exons had a median GC-content of 0.52 compared to high-GC content first exons’ median of 0.74 (**Figure S3**).

**Figure S3.** Box-plot of GC-content distribution in all first-exons (*blue*) and high-GC content (>70% GC; >=50 bp length).

We looked at a subset of bases that were targeted by the exome-capture kit and have high-GC and found that aggregate data recovers 38.7% +/- 2% compared to an average of 14.9 - 19.1% at 20x threshold with regular (typically averaged ~100X) WES sequencing in GC-rich first exons (Table S5). Only 50-60% of total targeted bases have bases mapped to them (≥*1X*; 570,000-640,000 / 894,578), suggesting mapping or capture issues. While there is approximately two-fold improvement (~19% to ~38%) in sequence interpretation in high GC-content exons due to aggregate sequencing, there is considerable necessity for improvement to target, capture and optimize sequencing of GC-rich exons.

**Table S5.** Evaluation of coverage of targeted exons with high GC content (attached excel file).

**Table S6.** Quote from Illumina for exome enrichment kits. Quotes in *red* indicate costs when the study was undertaken. *Nextera* prices, and other kit prices (in *white*) reflect current costs per sample (see last column).

**
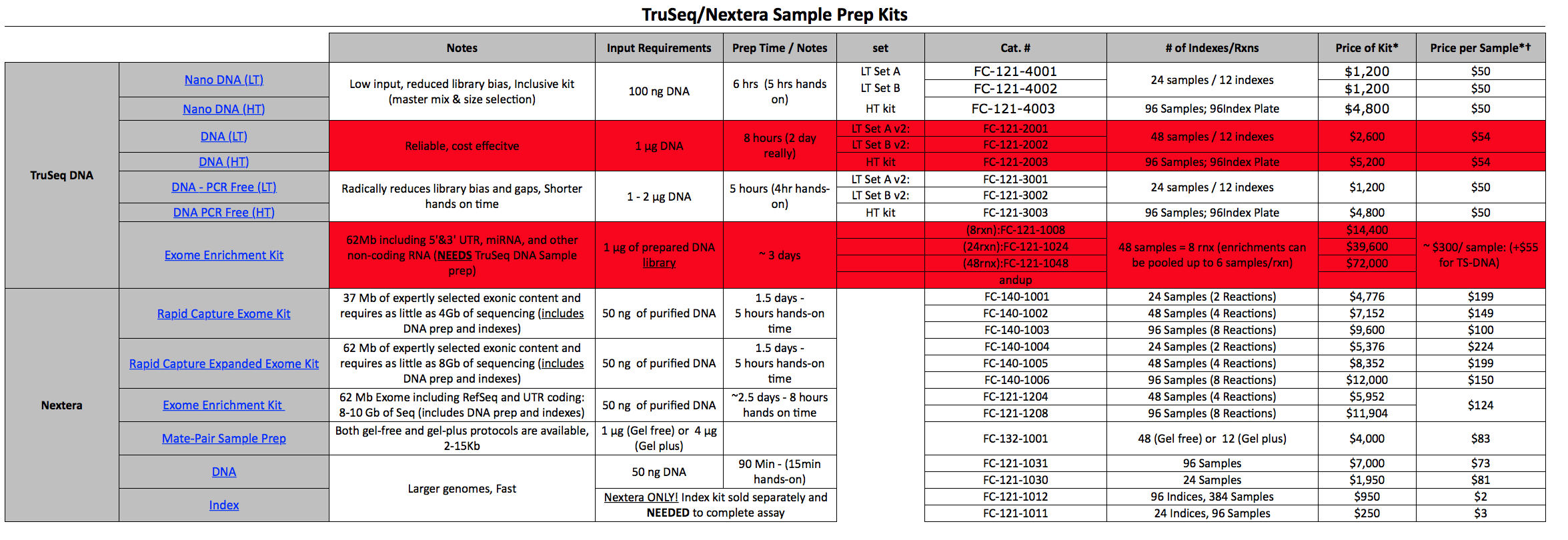
**
